# *Eucommia ulmoides* Leaf Polysaccharide in Conjugation with Ovalbumin Act as Delivery System Can Improve Immune Response

**DOI:** 10.3390/pharmaceutics13091384

**Published:** 2021-09-01

**Authors:** Haibo Feng, Jie Yang, Hui Zhi, Xin Hu, Yan Yang, Linzi Zhang, Qianqian Liu, Yangyang Feng, Daiyan Wu, Hangyu Li

**Affiliations:** 1 College of Animal Husbandry and Veterinary Medicine, Southwest Minzu University, Chengdu 610041, China; zlz754130837@163.com (L.Z.); 15892603728@163.com (Q.L.); f1733678933@126.com (Y.F.); wdx1063196822@126.com (D.W.); li1998hangyu@163.com (H.L.); 2 Key Laboratory of Ministry of Education and Sichuan Province for Qinghai-Tibetan Plateau Animal Genetic Resource Reservation and Utilization, Chengdu 610041, China; 3 Department of Veterinary Medicine, Southwest University, Chongqing 402460, China; yangyanswu@yahoo.com (J.Y.); slimrecovery@163.com (H.Z.); huxinvet@163.com (X.H.); yangyanxndx@163.com (Y.Y.)

**Keywords:** polysaccharides from *Eucommia ulmoides*, adjuvant, delivery system, macrophage, dendritic cell

## Abstract

In this investigation, to maximize the desired immunoenhancement effects of PsEUL and stimulate an efficient humoral and cellular immune response against an antigen, PsEUL and the model antigen ovalbumin (OVA) were coupled using the N-(3-dimethylaminopropyl)-N′-ethylcarbodiimide hydrochloride (EDC) reaction to yield a novel delivery system (PsEUL-OVA). The physicochemical characteristics and immune regulation effects of this new system were investigated. We found the yield of this EDC method to be 46.25%. In vitro, PsEUL-OVA (200 μg mL^−1^) could enhance macrophage proliferation and increase their phagocytic efficiency. In vivo, PsEUL-OVA could significantly increase the levels of OVA-specific antibody (IgG, IgG1, IgG2a, and IgG2b) titers and cytokine (IL-2, IL-4, IL-6, IFN-γ) levels. Additionally, it could activate T lymphocytes and facilitate the maturation of dendritic cells (DCs). These findings collectively suggested that PsEUL-OVA induced humoral and cellular immune responses by promoting the phagocytic activity of macrophages and DCs. Taken together, these results revealed that PsEUL-OVA had the potential to improve immune responses and provide a promising theoretical basis for the design of a novel delivery system.

## 1. Introduction

*Eucommia ulmoides Oliv*. (EU) has been used as traditional Chinese medicine for at least 2000 years and is commonly known as “Du-Zhong” in China. It is obtained from the stem bark of 15- to 20-year-old *Eucommia ulmoides Oliv*. trees and was recorded in ancient Chinese medical texts as “Shennong’s Herba”. This ancient nourishing herb was commonly used as a tonic to strengthen bones and muscles and nourish the liver and kidneys. This herb was also used as a diuretic and as a treatment for a variety of pathological conditions, including arthritis, recurrent miscarriage, rheumatism, and lumbago [1] Polysaccharides extracted from EU (PsEUL) have been widely studied in recent years as a pharmaceutical component owing to its immunomodulatory effect [2,3]. According to previous reports, the average molecular weight (*Mw*) of *Eucommia ulmoides Oliv*. Polysaccharides (EUPS) was determined to be 11.4632 × 10^5^ Da. The monosaccharide components of EUPS are glucose, fructose, mannose, fucose, galactose, and arabinose, with a relative mass of 36.6%, 16.6%, 14.2%, 15.7%, 9.5%, and 7.4%, respectively [4]. Due to the shortage of EU bark resources, in order to make full use of EU leaf resources, studies on EU have focused mainly on the effective components and pharmacological activities of EU leaves. The leaves of EU are commonly used as a nourishing tonic [4]. Extracts from EU leaves demonstrate antioxidant and anti-atherosclerotic effects, and were found to increase piglet growth performance and decrease rates of diarrhea [5].

Eliciting an immune response is the most effective way for the body to fight harmful substances. Currently, naturally occurring polymer antigens, such as proteins, are commonly used [6], and these are pharmacologically active and have high safety margins [7]. However, there are also problems associated with natural proteins, for example, poor drug stability and a short half-life, among other shortcomings [8], especially during the immune response. There are many antigen-recognition receptors on the surface of immune cells; however, not every natural antigenic protein has a corresponding antigen-recognition receptor [9]. Therefore, attempts are being made to modify the structure of antigenic proteins to improve their immune effects [10].

Naturally occurring macromolecules, especially plant polysaccharides, have immunomodulatory effects on the body owing to their unique structural properties [11,12,13]. Polysaccharide-modified antigenic proteins can enhance their recognition as antigens by immune cells without changing the reaction specificity that is elicited toward natural antigens [14]. The binding receptors of polysaccharides on the surface of immune cells are a kind of pattern-recognition receptor [15]. Polysaccharide, which is an important ligand for these receptors, can further help immune cells to recognize antigens and enhance the body’s immune response [16]. β-glucan can promote cellular and humoral immunity, whereas macrophages can be activated by binding to specific receptors such as CR-3 and Dectin-1 [17,18], which can affect the release of cytokines. Therefore, β-glucan has become a hot topic in the study of polysaccharide-binding proteins [19]. Conversely, there is significant research being conducting regarding the use of proteins to improve the properties of polysaccharides, such as in meningitis caused by *Neisseria meningitidis* and pneumonia by *Streptococcus pneumoniae*. These conditions have a common causative factor, i.e., the bacterial capsular polysaccharide [20,21]. Although vaccines produced using capsular polysaccharides as antigens are available, most of them have the drawback of an inability to provide immunological protection to infants and children; therefore, studies have been carried out to covalently combine capsular polysaccharides with carrier proteins to produce vaccines that can effectively protect this younger population [22,23,24].

In general, the use of polysaccharide-binding proteins is one of the ways for these treatments to complement each other, whether they are protein- or polysaccharide-based, as the primary aim is to improve drug delivery. Many researchers have focused their attention on coupling proteins with polysaccharides using the EDC condensation reaction. EDC activates proteins and the activated proteins are chemically bonded to the polysaccharides; these synthetic products can retain the activity of proteins and polysaccharides to the greatest extent while being absorbed by the body to elicit a pharmacological response [25,26].

In this study, we conjugated PsEUL with OVA for the first time, using the EDC method to synthesize an antigen delivery system (PsEUL-OVA) that uses PsEUL to assist OVA and hypothesized that the specific immune response of antigens could be increased using this method. We evaluated the immune response of PsEUL-OVA in vitro and in vivo. The aim of this investigation was to evaluate the use of the EDC method to conjugate PsEUL and OVA to improve their immune-enhancing activity, thus providing crucial application prospects in drug delivery system development.

## 2. Materials and Methods

### 2.1. Materials

*Eucommia ulmoides Oliver* leaf was obtained from SPH Zunyi Pharmaceutical Co., Ltd. (Zunyi, China). OVA and Freund’s Adjuvant, Complete, was purchased from Sigma-Aldrich Co., LLC (St Louis, MO, USA). EDC and ADH were purchased from Shanghai Aladdin Biochemical Technology Co., Ltd. (Shanghai, China). Sephadex G-150 was obtained from Shanghai Yuanye Bio-Technology Co., Ltd. (Shanghai, China). Cell Counting Kit-8 (CCK-8) was purchased from Shanghai Beyotime Biotechnology Co., Ltd. (Shanghai, China). The mouse macrophage (RAW264.7) cell line was obtained from Shanghai Zhong Qiao Xin Zhou Biotechnology Co., Ltd. (Shanghai, China). Phalloidin-iFluor 555 Reagent was obtained from Abcam Inc. (Abcam, Cambridge, MA, USA) and 4′,6-diamidino-2-phenylindole (DAPI) was obtained from Solarbio (Beijing, China). HRP goat anti-mouse IgG, IgG1, IgG2a, and IgG2b were obtained from Santa Cruz Biotechnology, Inc. (Santa Cruz, CA, USA). IL-2, IL-4, IL-6, and an IFN-γ mouse uncoated enzyme-linked immunosorbent assay (ELISA) kit were obtained from Thermo Fisher Scientific Inc. (Asheville, NC, USA).

### 2.2. Preparation of PsEUL

PsEUL was obtained via hot water extraction and alcohol precipitation [27]. *E. ulmoides* leaf powder was ground and passed through a 60-mesh sieve. Next, the sieved leaf powder was mixed in a ratio of 1:20 with distilled water and was subjected to ultrasonication in a water bath at 60 °C for 2 h. The filtrate was evaporated under reduced pressure and the protein was removed using the Sevag method [28] by treating it with 95% ethanol overnight at 4 °C to obtain PsEUL. The content of polysaccharides was determined using the phenol-sulfuric acid method [29].

### 2.3. Coupling of Polysaccharides and Proteins

**Figure 1:**
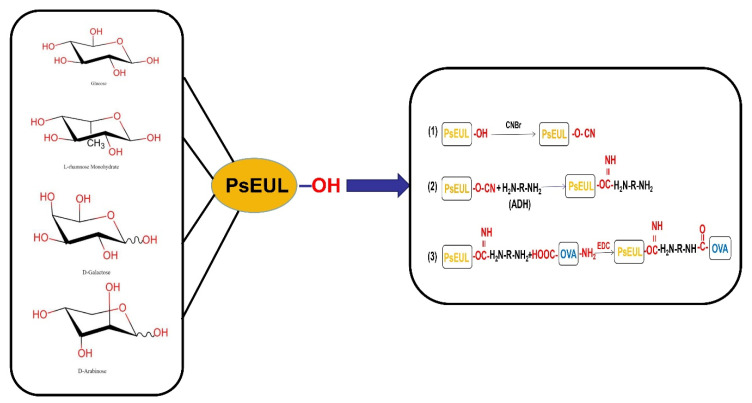
Schematic diagram of preparing the mannose modified carbon nanotubes antigen delivery system.

About 800 mg PsEUL was dissolved in 200 mL sodium chloride solution (0.15 M) and reacted with cyanogen bromide at 20 °C for 15 min (pH 10.8). Then, 200 mL of ADH solution was added and the pH of the reaction mixture was adjusted to 8.6 using 0.5 M hydrochloric acid solution; the mixture was left at 20 °C overnight. The next day, the solution was dialyzed (10 kDa) against 20 mM EMS buffer to remove excess ADH and cyanogen bromide. This process is called polysaccharide activation [30]. A volume of 200 mL activated PsEUL solution was mixed with OVA (4 mg/mL) and 15 mL EDC added and reacted at 20 °C for 12 h. Then, the excess EDC was removed using dialysis bags (10 kDa) at 4 °C in a 0.15 M sodium chloride system to obtain the coupling product of PsEUL and OVA (Figure 1).

Separation and purification of PsEUL-binding OVA (PsEUL-OVA) were performed using column chromatography and the column volume (1.2 × 30 cm) was 300 mL. The sample was loaded on a Sephadex G-150 column equilibrated with PBS. After loading, the sample was eluted using a flow rate of 1 mL/min for the PBS (pH 7.4) solution. The eluent was collected using an automatic collection device (BSZ-160F, Shanghaijingke Co., Ltd., Shanghai, China) at a rate of one tube per minute using a Biuret kit. The collected fractions were detected at a wavelength of 540 nm and the elution curve was drawn. The separated and purified binding proteins were freeze-dried and the resultant powder was stored at 4 °C [31].

### 2.4. Structure Characterization

#### 2.4.1. FT-IR Spectrum

PsEUL and PsEUL-OVA were measured using Fourier transform infrared spectrometry (Nicolet/is5010400, Thermo Fisher Scientific, Asheville, NC, USA). Samples (1–2 mg) were mixed with potassium bromide and ground thoroughly. The powder was pressed into sheets using a tablet press and scanned in the band of 4000–400 cm^−1^ [32].

#### 2.4.2. Sodium Dodecyl Sulfate-Polyacrylamide Gel Electrophoresis (SDS-PAGE)

PsEUL-OVA was identified using SDS-PAGE in a discontinuous buffer system consisting of 5% stacking gel and 12% separating gel. The sample was dissolved in distilled water, SDS-PAGE sample loading buffer (4:1) was added, and each lane was loaded with 10 μL for electrophoresis. After electrophoresis, the gel block was stained using Coomassie Blue staining solution for 1 h and decolorized using Coomassie Blue destaining solution for 2 h. Protein markers ranging from 10–150 kDa were used as standards [33].

#### 2.4.3. SEM Analysis

Appropriate amounts of PsEUL and PsEUL-OVA were taken and adhered to the sample table using copper tape. The sample table was placed on an ion sputtering instrument, coated with a layer of conductive gold powder, and was placed under the SEM system (JEOL, JSM-7500F, Tokyo, Japan) and observed. Each sample was photographed three times to eliminate sample interference and system errors [34].

#### 2.4.4. Analysis Using Laser Particle-Size Analyzer

PsEUL, OVA, and PsEUL-OVA were dissolved in distilled water, the solution was added to a quartz cuvette, and the particle size was measured using a laser particle-size analyzer (Litesizer 500, Anton-Paar, Vienna, Austria) [35].

#### 2.4.5. Zeta Potential Analysis

PsEUL, OVA, and PsEUL-OVA were dissolved in distilled water, the solution was added to a disposable folded capillary sample cell. The samples were analyzed using a laser particle size analyzer (Litesizer 500, Anton-paar, Vienna, Austria) to measure its zeta potential [35].

#### 2.4.6. Polysaccharides and Protein Binding Degree

PsEUL-OVA was separated using column chromatography and purified. The BCA method [36] was used to draw a standard curve using OVA as the protein standard to determine the protein content of samples. Using the standard curve, the quality of PsEUL-OVA was determined after separation and purification. The phenol sulfate method was used and the quality of the polysaccharide in the polysaccharide-binding protein was obtained using the following formula:

Polysaccharide-binding protein conversion rate (100%) = $\;\frac{P+C}{M}$ × 100%, the ratio of polysaccharide-binding protein to polysaccharide is as follows: in the polysaccharide–protein ratio = $\frac{P}{C}$, P is the quantity of OVA in the polysaccharide-binding protein after separation and purification. C is the quantity of polysaccharide in the polysaccharide-binding protein. M is the total dose.

### 2.5. Macrophage Activity and Phagocytosis Test

After adjusting the cell concentration of macrophages, they were placed in a 96-well cell-culture plate. After 24 h of culture, different concentrations of polysaccharide-binding protein solution at 50, 100, 150, 200, 250, 300, and 350 μg/mL were added and cultured for a further 24 h. Next, 10 μL of Cell Counting Kit-8 (CCK-8) reagent was added to each well and the OD was determined using a microplate reader at 450 nm after 4 h. Based on the OD value, the most suitable drug concentration was calculated using the following cell activity formula, as stated in the CCK-8 kit:

(1) $$\mathrm{Cell}\;\mathrm{viability}\;\left(\%\right)=\frac{\left(A_{S}-A_{B}\right)}{\left(A_{Z}-A_{B}\right)}\times 100\%$$

(A_S_: with cells, CCK-8 solution absorbance of drug wells; A_B_: absorbance of wells with medium and CCK-8 solution without cells; A_Z_: absorbance of wells with cells and CCK-8 solution without drugs).

The PsEUL-OVA was mixed with fluorescein isothiocyanate (FITC) in dimethyl sulfoxide (DMSO) at 4 °C for 12 h, dialyzed against PBS at 4 °C for 72 h, and lyophilized into a powder to obtain FITC-OVA-PsEUL. A similar method was used to prepare FITC-OVA. Macrophages were inoculated in a 6-well cell-culture plate with a round coverslip. After 24 h of culture, FITC-OVA-PsEUL and FITC-OVA were added; after a further incubation of 12 h, the slides were taken out and fixed with 4% paraformaldehyde for 20 min and permeabilized with Triton X-100 for 4 min. Next, they were stained with Phalloidin-iFluor 555 Reagent for 50 min and with DAPI staining solution for 5 min. Lastly, the macrophages were mounted with 90% glycerol and photographed using a confocal laser scanning microscope (LSM 800, ZEISS, Oberkochen, Germany).

### 2.6. Immunization Grouping and Processing of Serum and Immune Organs

Female ICR mice (Grade II, weight: 18–22 g) were provided by Beijing Charles River Laboratory Animal Technology Co., Ltd., (Beijing, China). All animal procedures were performed as per internationally accepted principles mentioned in the Guidelines for Keeping Experimental Animals issued by the Government of China. The protocols were approved by the IACUC, Southwest University (NO: IACUC-20191223-16). Ninety 4-week-old female ICR mice were selected and randomly divided into 6 groups of 15 mice each. The first immunization was performed after one week of adaptive feeding. Mice in the blank group were injected subcutaneously with 0.2 mL of saline. The second group comprised the PsEUL group and mice were injected with 0.2 mL (500 μg/mL) PsEUL solution. The third group was the OVA group and mice were injected with 0.2 mL (500 μg/mL) OVA solution. In the PsEUL + OVA group, mice were injected with 0.2 mL PsEUL and 0.2 mL OVA (500 μg/mL) solution. PsEUL-OVA constituted the fifth group and mice were injected with 0.2 mL (1000 μg/mL) PsEUL-OVA solution. The sixth group was a positive control group and mice were injected with 0.2 mL OVA (500 μg/mL) in FCA and a distilled water mixed solution (1:1). A booster immunization was carried out once a week and a total of four immunizations were performed. Each immunization dose was similar to that of the first immunization (3 days after the first immunization, 3 mice were randomly selected from each group and their spleens were collected for flow cytometry analysis).

One week after the first immunization, 3 mice were randomly selected from each group for blood collection; the serum was separated and stored in a −20 °C refrigerator. The thymus and spleen were collected to calculate the immune organ index. The above procedures were performed every week thereafter throughout the duration of the immunization program. The mouse immune organ index was calculated using the following formula:

(2) $$\mathrm{Immune}\;\mathrm{organ}\;\mathrm{index}\;\left(\mathrm{mg}/g\right)=\frac{\mathrm{Weight}\;\mathrm{of}\;\mathrm{immune}\;\mathrm{organ}\;\left(\mathrm{mg}\right)}{\;\mathrm{Body}\;\mathrm{weight}\;\left(g\right)}\times 100\%$$

where immune organ index (mg/g) = weight of immune organ (mg)/body weight (g).

### 2.7. Flow Cytometry (FCM) Analysis

After 3 and 28 d following the first immunization, 3 mice were randomly selected from each group for flow cytometry. Their spleens were collected and ground to make a single-cell suspension. PE and FITC double staining methods were used; the cell surface staining markers included CD3, CD4, CD8, CD11c, MHC-II, CD80, and CD86. The double-stained cell suspensions were transferred to a flow cytometer tube and analyzed using flow cytometry (BD FACSVerseTM, BD Biosciences, San Jose, CA, USA).

### 2.8. Serum Antibody Level Determination

The OVA-specific IgG and IgG subtypes (IgG1, IgG2a, and IgG2b) in serum samples were detected using ELISA. Briefly, the coating solution containing OVA was added to the 96-well ELISA plate for 18 h. After three washes, 5% of skim milk was added to the block and incubated at 37 °C for 1 h. After three washes, horseradish peroxidase (HRP)-labeled goat anti-mouse antibody (IgG and IgG1, IgG2a, IgG2b) was added to each well and incubated for 1 h at 37 °C. Lastly, the serum samples were added to the 96-well plate and incubated at 37 °C for 1 h. After washing, the substrate was developed using 3,3′,5,5′-tetramethylbenzidine (TMB) and then the reaction was terminated by adding 2 M H_2_SO_4_. The OD at 450 nm was measured using a microplate reader (Bio-Rad, iMark, Hercules, CA, USA).

### 2.9. Serum Cytokine Analysis

Cytokines IL-2, IL-4, IL-6, and IFN-γ in mouse serum were measured using an ELISA kit and a microplate reader was used to measure the OD at 450 nm.

### 2.10. Statistical Analysis

Test results are expressed as the mean ± standard deviation. SPSS 22 software (SPSS, Chicago, IL, USA) was used for data analysis. Duncan and LSD multiple range tests were used to determine the differences among groups. Different letters (a, b, c, d, and e) above each group indicate statistically significant differences (*p* < 0.05). The order of statistically different activities in letters is as follows a > b > c > d > e.

## 3. Results

### 3.1. Physicochemical Characteristics of PsEUL

PsEUL was obtained as a brown powder, soluble in water. Although it partially precipitated in water, it could be completely dissolved by heating. The total sugar content was assessed using the phenol sulfate method and determined to be 96.36%. The yield of PsEUL was calculated as 5.7%. In accordance with previous reports, the monosaccharide composition of PsEUL was analyzed using high-performance liquid chromatography (HPLC). The level of glucose was highest and found to be between 38.2% and 39.1%, followed by L-arabinose (37.7%), D-galactose (12.8%), and L-rhamnose monohydrate (11.8%). Alduronic acid accounted for 21.2% of the important components of PsEUL. The molecular weight of PsEUL ranged from 6 × 10^4^–6 × 10^5^ Da [37,38]. This indicated that PsEUL is a polysaccharide with high molecular weight and conforms to the previous description that PsEUL will show partial precipitation in water.

### 3.2. Separation and Purification of PsEUL-OVA

**Figure 2:**
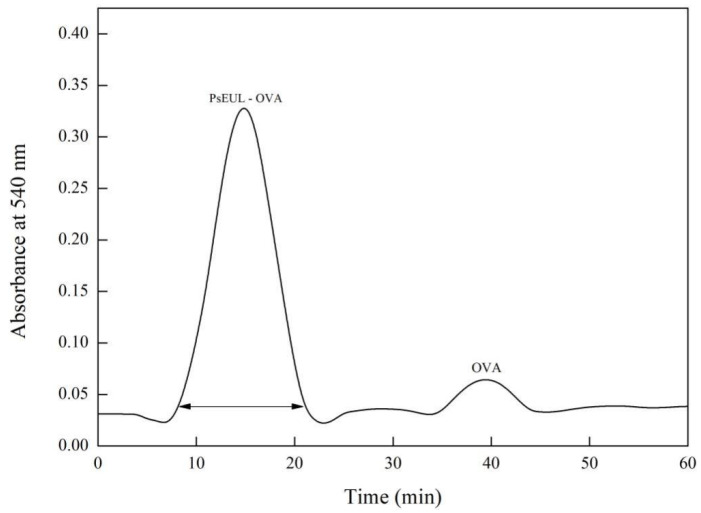
Elution curve of PsEUL-OVA.

Sephadex G-150 column chromatography was used to purify PsEUL-OVA [39,40]. The maximum absorption wavelength of the colored solution in the ultraviolet-visible spectrum is 540 nm [41]. The OD of samples treated with Biuret reagent was measured at 540 nm and an elution curve was drawn. As illustrated in Figure 2, PsEUL-OVA was eluted as the main peak from the Sephadex G-150 column. The elution position is shown by an arrow. The PsEUL-OVA showed a relatively symmetrical single peak at 8–22 min; a relatively small single peak also appeared at 30 min, indicating that PsEUL was successfully coupled to OVA to form PsEUL-OVA with a larger molecular weight, which was eluted first. The small peak at 30 min represented the OVA that was not fully coupled. Because its molecular weight is lower than that of PsEUL-OVA, it had a longer path through the Sephadex G-150 column before being finally eluted. It can be seen from the comparison of the area sizes of the two peaks that the PsEUL-OVA generated via the EDC-shrinkage method was highly efficient and only a small part of OVA did not bind to the PsEUL. The purified PsEUL-OVA was obtained by collecting the eluent for 8–22 min and concentrating it in an ultrafiltration tube.

### 3.3. Structure Characterization

#### 3.3.1. FT-IR Spectrum

**Figure 3:**
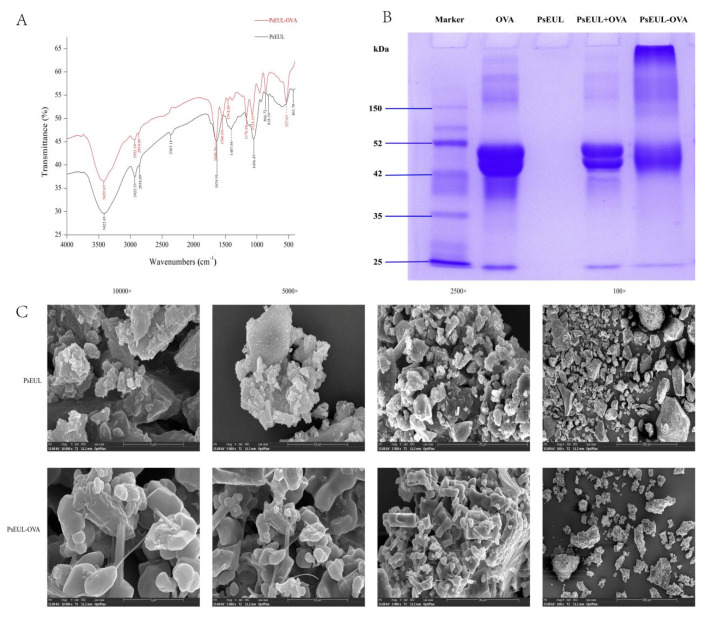

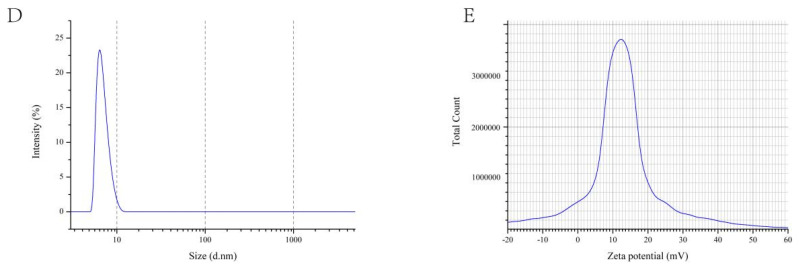
Physicochemical characterization of PsEUL and PsEUL-OVA. FT-IR spectrum of PsEUL and PsEUL-OVA (**A**); SDS-PAGE profile of OVA and PsEUL-OVA (**B**); scanning electron micrographs of PsEUL and PsEUL-OVA (**C**); particle size (**D**) and zeta potential (**E**) of PsEUL-OVA.

FT-IR was used to scan PsEUL and PsEUL-OVA in the band of 4000–400 cm^−1^, and the results are shown in Figure 3A. The PsEUL has a typical polysaccharide absorption peak, which indicates O-H stretching vibration at 3422 cm^−1^ [42]. At 2922 cm^−1^, C-H stretching vibration of CH_3_, CH_2_, and CH was observed [43]. At 1654 cm^−1^, the stretching vibration of the amide group C=O at 1056 cm^−1^ and 461 cm^-1^ indicated the presence of the pyranose ring. At 866 cm^−1^, the variable angle vibration of CH in the β-pyran ring, at 818 cm^−1^ was observed, indicating the vibration absorption peak of the C-H variable angle in the furan ring [44]. Based on FT-IR characteristics, it could be inferred that PsEUL is an acidic polysaccharide, comprising pyranose and furan rings. The infrared spectra of PsEUL-OVA and PsEUL were compared and the stretching vibration peak of PsEUL-OVA at the position of O-H at 3422 cm^−1^ was lower than that of PsEUL. This finding indicated that the O-H of PsEUL was activated by CNBr and corresponded to the variable-angle vibration absorption peak of NH at 1514 cm^−1^ [45]. 1124 cm^−1^ corresponded to the CN stretching vibration in EDC [42]. At the same time, the infrared spectrum of the PsEUL-OVA showed a corresponding C=O stretching vibration peak on adipic acid hydrazide at 1580 cm^−1^, indicating that the PsEUL had succeeded in coupling to OVA through the formation of a covalent bond (-CO-NH-).

#### 3.3.2. SDS-PAGE

It is well known that the condensation reaction using EDC results in an increase in the molecular weight of the protein. The molecular weight of OVA is 44.5 kDa. Previous studies have reported that the molecular weight of PsEUL is between 60 and 600 kDa; after the coupling of PsEUL and OVA, the molecular weight of PsEUL-OVA would, therefore, be significantly higher than that of OVA. As shown in Figure 3B, OVA and PsEUL-OVA were detected using SDS-PAGE. Compared to OVA, the band of PsEUL-OVA moved up to the compressed rubber part, indicating that the molecular weight had increased, which showed the successful coupling of PsEUL with OVA. The band of PsEUL-OVA had reached the top; therefore, the molecular weight of PsEUL-OVA could not be accurately detected using electrophoresis. This was mainly because the molecular weight of PsEUL was much greater than 12% of the maximum molecular weight that SDS-PAGE could bear and consequently, PsEUL-OVA was unable to enter the separation gel. It can also be seen in Figure 3B that a part of PsEUL-OVA appears on the top of the separation gel. This finding can be explained based on the fact that PsEUL is a mixture of multiple low molecular weight polysaccharides. The PsEUL-OVA formed through the combinination with OVA could enter the separation gel; however, because the molecular weight of this part of the PsEUL-OVA was still much larger than that of OVA, it remained on the top of the separation gel [46,47,48].

#### 3.3.3. SEM Analysis

It can be seen from the SEM images at different magnifications that PsEUL appears as a relatively smooth and dense block at 100 times, whereas a large number of fine particles are attached to the surface of PsEUL-OVA. At a higher magnification, it can be seen that the surface of PsEUL-OVA is smoother than that of PsEUL and there is a clear connection between the particles. In general, SEM images indicate that the structure of PsEUL and PsEUL-OVA has changed significantly. The images also indicate that PsEUL-OVA exhibits the adhesion of objects, thereby depicting the successful combination of OVA and PsEUL (Figure 3C).

#### 3.3.4. Laser Particle Size Tester

Previous analysis shows that PsEUL-OVA has a relatively large molecular weight. Studies have confirmed that the size of the drug is related to its systemic bioavailability [49]. Therefore, it is important to understand the particle size and stability of the PsEUL-OVA in the liquid. The particle size and zeta potential of PsEUL-OVA were detected using a laser particle-size analyzer (Figure 3D,E). The average particle size of PsEUL-OVA was determined to be 8.98 ± 2.39 nm, the polymer dispersity index (PDI) to be 0.081 ± 0.01, and the average zeta potential to be 15.49 ± 0.3 mV, revealing that the PsEUL-OVA had good dispersion and was stable.

#### 3.3.5. Polysaccharides and Protein Binding Degree

The conversion rate of polysaccharide-binding protein (%) = 46.25% calculated using the BCA and phenol sulfate methods. The ratio of polysaccharide to protein was determined to be 12:25.

### 3.4. In Vitro Tests

#### 3.4.1. Cytotoxicity Analysis of PsEUL-OVA on RAW264.7

**Figure 4:**
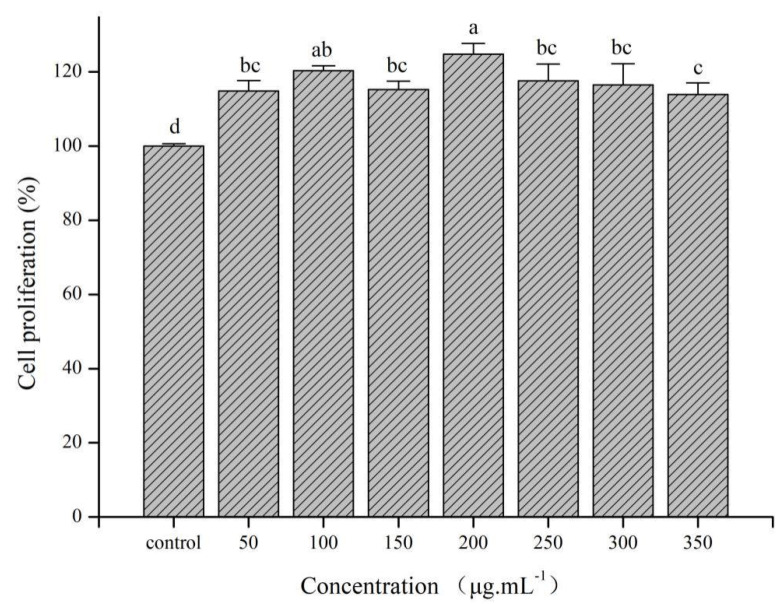
Effect of PsEUL-OVA on the proliferation of macrophages. The proliferation activity was measured using the CCK-8 method. The values are presented as mean ± SD (*n* = 3). Different letters (a–d) above each group of bars indicate statistically significant differences (*p* < 0.05).

As important immune regulatory cells of the immune system, macrophages not only participate in eliciting specific and non-specific immune responses but also serve as “bridge cells” between the two [50]. Therefore, their normal function directly or indirectly affects the possibility of antigen presentation and clearance during an immune response. The immune-modulation effect of PsEUL-OVA on macrophages is shown in Figure 4. The PsEUL-OVA was not cytotoxic to macrophages in the concentration range of 0–350 μg mL^−1^ and even increased cell proliferation. The maximum cell proliferation activity was observed at a concentration of 200 μg mL^−1^ and was significantly different from the blank control group (*p* < 0.05). Therefore, a concentration of 200 μg mL^−1^ was selected for subsequent experiments.

#### 3.4.2. Effects of PsEUL-OVA on the Phagocytic Activity of Macrophages

**Figure 5:**
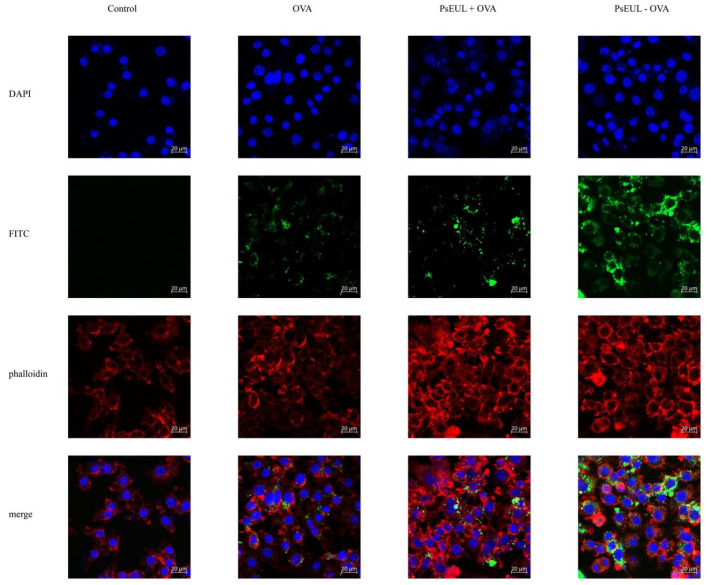
Laser confocal scanning microscopy of PsEUL-OVA uptake by macrophages. Macrophages were inoculated in a 6-well cell-culture plate with a round coverslip. After 24 h of culture, OVA, FITC-OVA-PsEUL, and FITC-OVA were added separately. After incubation for 12 h, the slides were taken out and fixed and stained using DAPI and Phalloidin-iFluor 555. Blue fluorescence indicates the nucleus, labeled by DAPI, whereas red fluorescence indicates the actin stained with Phalloidin-iFluor 555. Cells were mounted with 90% glycerol and photographed using a confocal laser scanning microscope.

In order to explore the ability of macrophages to take up PsEUL-OVA, the phagocytic effect of macrophages on antigens was observed using laser confocal scanning microscopy. Different fluorescence intensities indicate the uptake of different antigens by macrophages. It can be seen in Figure 5 that the fluorescence intensities of the PsEUL-OVA and PsEUL+OVA groups are significantly higher than that of the OVA group. This shows that PsEUL could enhance the ability of macrophages to take up antigens. Compared to the PsEUL+OVA group, the fluorescence intensity of the PsEUL-OVA group was significantly increased. We found that PsEUL-OVA was mainly distributed in the cytoplasm and there was also a large amount of antigen adsorbed on the cell surface to be phagocytosed by macrophages. These findings indicated that the coupling of PsEUL and OVA using the EDC method could induce macrophages to take up more OVA.

### 3.5. Effect of PsEUL-OVA on Immune Organ Index in Mice

**Figure 6:**
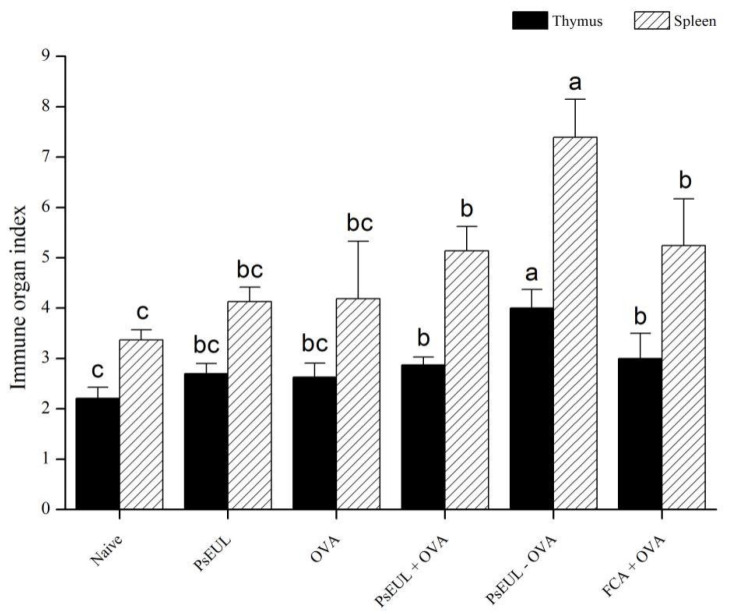
Effect of PsEUL-OVA on the organ index of ICR mice. The thymus and spleen indices of mice were calculated using the following equation: organ index (mg/g) = (weight of the immune organ (mg)/body weight (g)) × 100%. Results are presented as mean ± SD (*n* = 3). Different letters (a–c) above each group of bars indicate statistically significant differences (*p* < 0.05).

The thymus and spleen are important immune organs, responsible for lymphocyte production [51]. When the body is attacked by foreign objects, an immune reaction occurs, in which a large number of B lymphocytes and T lymphocytes are produced. Therefore, the thymus and spleen are indicative of appropriate compensatory growth and determining the immune organ index can reflect the immune function of the body. The thymus and spleen indices of mice are shown in Figure 6. In comparison with the naive group, the immune organ index of other groups had significantly increased (*p* < 0.05). The organ index in the PsEUL-OVA group was significantly higher than that of the FCA+OVA group (*p* < 0.05). Our results demonstrate that PsEUL-OVA could promote the immune organ index.

### 3.6. Effect of PsEUL-OVA on the OVA-Specific IgG and the IgG Subclasses in Mouse Serum

**Figure 7:**
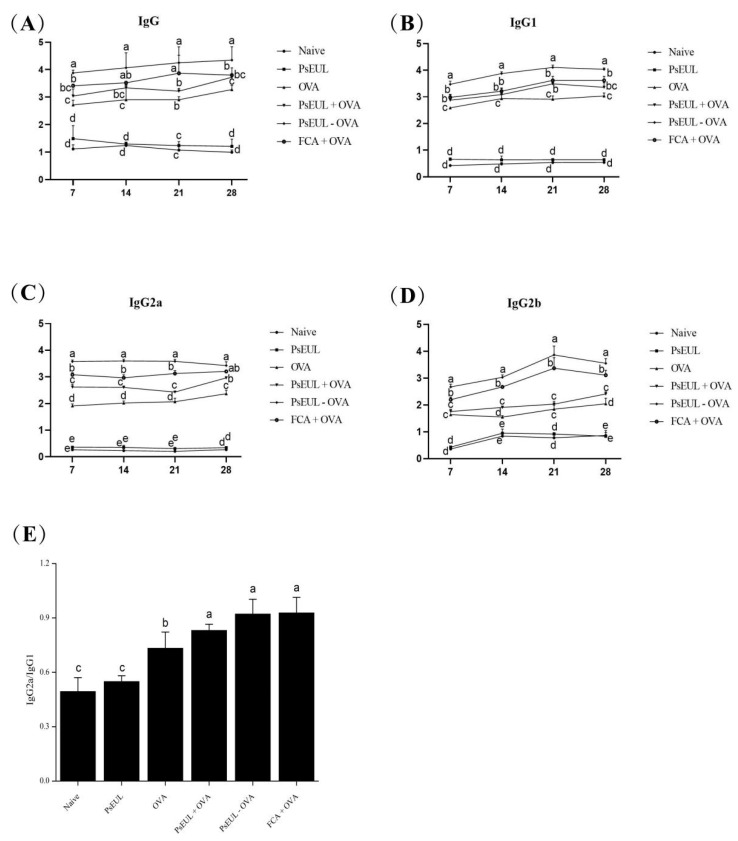
Effects of PsEUL-OVA on antibody response. Serum samples were collected for ELISA from immunized ICR mice of all groups on day 14 after the second immunization. Serum samples of ICR mice collected 7–28 days after the first immunization. OVA-specific IgG (**A**), OVA-specific IgG subtypes IgG1 (**B**), IgG2a (**C**), and IgG2b (**D**). IgG2a/IgG1 ratio (**E**) were analyzed using ELISA. Results are presented as mean ± SD (*n* = 3). Bars marked with different letters (a–e) indicate statistically significant differences (*p* < 0.05).

We explored the effect of PsEUL-OVA on the humoral immune response of mice. OVA-specific IgG and IgG subtypes in mouse serum were measured using ELISA on days 7, 14, 21, and 28 after the first immunization. Results of OVA-specific IgG antibodies in mouse serum are shown in Figure 7A. The PsEUL-OVA and FCA+OVA groups produced significantly higher levels of specific IgG than the OVA group (*p* < 0.05), whereas the PsEUL+OVA group produced specific IgG and no significant difference was observed compared to the OVA group (*p* > 0.05). Among them, the specific IgG produced by the PsEUL-OVA group was significantly higher than that in the FCA+OVA group on days 7 and 28. There was no significant difference between the specific IgG produced by the PsEUL-OVA and FCA+OVA groups on days14 and 21 (*p* < 0.05). Our results showed that PsEUL-OVA could induce a high level of OVA-specific IgG and indicated a positive correlation between antibody levels and the number of immunizations.

OVA-specific subtypes in mouse serum are illustrated in Figure 7B. The OVA-specific IgG1 levels in the PsEUL-OVA and FCA+OVA groups were found to be significantly higher than those in the OVA group, in which the IgG1 produced in the PsEUL-OVA group at various time points was significantly higher than that in the FCA+OVA group (*p* < 0.05). As illustrated in Figure 7C, IgG2a levels in the PsEUL-OVA, PsEUL+OVA, and FCA+OVA groups were significantly higher than those in the OVA group (*p* < 0.05). The IgG2a in the PsEUL-OVA group was significantly higher than that of the FCA+OVA group on days 7, 14, and 21 (*p* < 0.05) and there was no significant difference between the PsEUL-OVA and FCA+OVA groups on day 28 (*p* > 0.05). It was observed that the amount of IgG2a produced by mice immunized with PsEUL-OVA was not directly related to the number of immunizations. The levels of IgG2b in the PsEUL-OVA and FCA+OVA groups were significantly higher than that in the OVA and PsEUL groups (Figure 7D, *p* < 0.05). The IgG2b antibody titer produced by the PsEUL-OVA group was significantly higher than that of the FCA+OVA group (*p* < 0.05), whereas the amount of IgG2b produced by the PsEUL-OVA group on day 21 was highest. This finding suggested that PsEUL-OVA could dramatically upregulate the OVA-specific subtype antibody titers.

As shown in Figure 7E, the ratios of IgG2a/IgG1 in the PsEUL+OVA, PsEUL-OVA, and FCA+OVA groups were significantly higher than that in the OVA group. There was no significant difference between the three groups. The ratio of IgG2a/IgG1 indicated that PsEUL-OVA could induce a Th1-biased immune response [52].

### 3.7. Effect of PsEUL-OVA on Serum Cytokines in Mice

**Figure 8:**
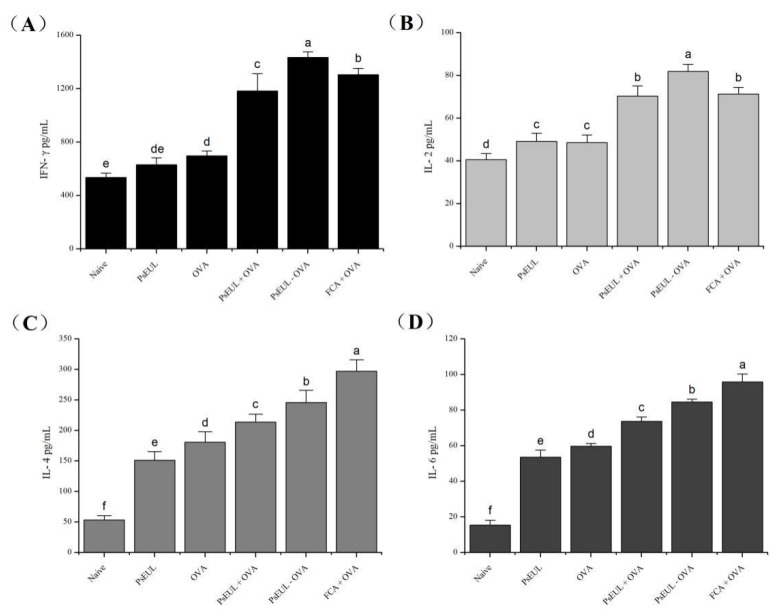
Effects of PsEUL-OVA on the serum cytokines concentration of mice. Serum samples of ICR mice collected on 28 days after the first immunization. Concentrations of IFN-γ (**A**), IL-2 (**B**), IL-4 (**C**), and IL-6 (**D**) in ICR mice serum were measured using ELISA. Results are presented as mean ± SD (*n* = 3). Different letters (a–f) above each group of bars indicate statistically significant differences (*p* < 0.05).

Cytokines are a class of protein molecules produced by immunocompetent cells. They play a very important role in the body’s immune regulation process [53]. IFN-γ and IL-2 are mainly secreted by Th1-type cells, whereas IL-4 and IL-6 are mainly secreted by Th2-type cells. The body’s immune function and the type of immune response can be evaluated by measuring cytokine levels. Cytokine levels in mouse serum are shown in Figure 8A,B. The levels of IFN-γ and IL-2 produced in the PsEUL-OVA group were significantly higher than those produced in other groups (*p* < 0.05). The IL-4 and IL-6 concentrations in mice, especially in the PsEUL-OVA group were significantly higher than those in the blank and OVA groups (*p* < 0.05, Figure 8C,D). Our results indicated that PsEUL-OVA could induce higher levels of Th1 and Th2 cytokine secretion, suggesting that it could enhance the balance of Th1/Th2 in the immune response.

### 3.8. Effect of PsEUL-OVA on Mouse T lymphocytes

**Figure 9:**
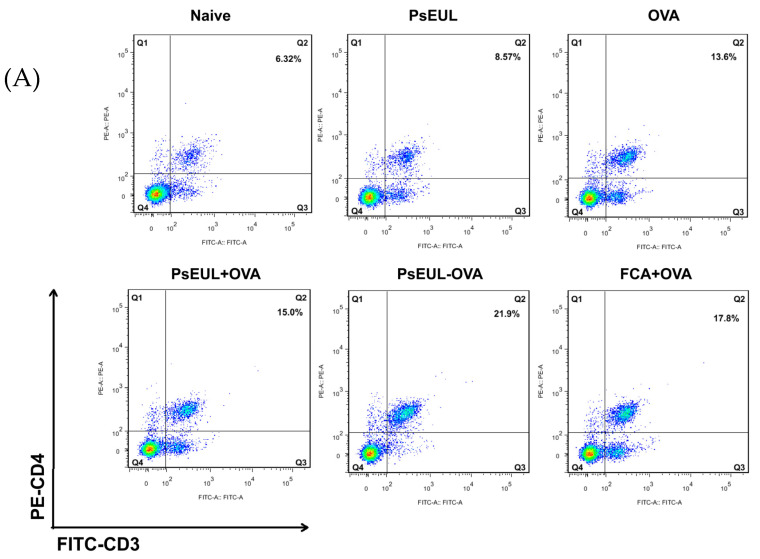

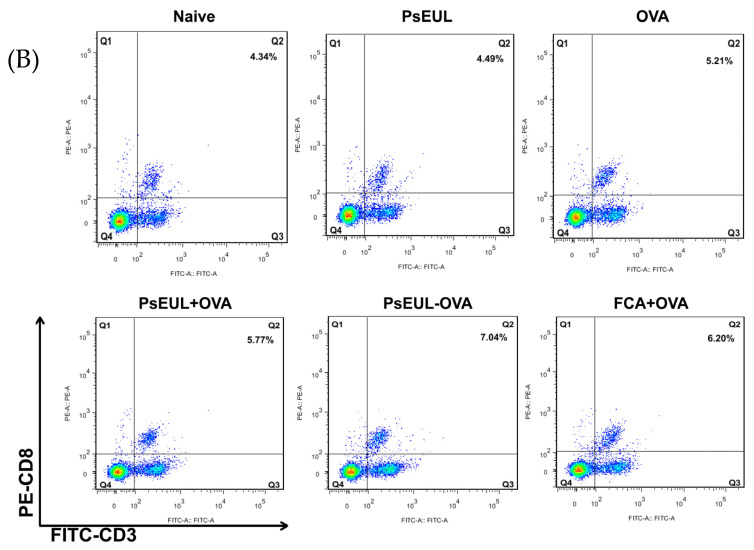

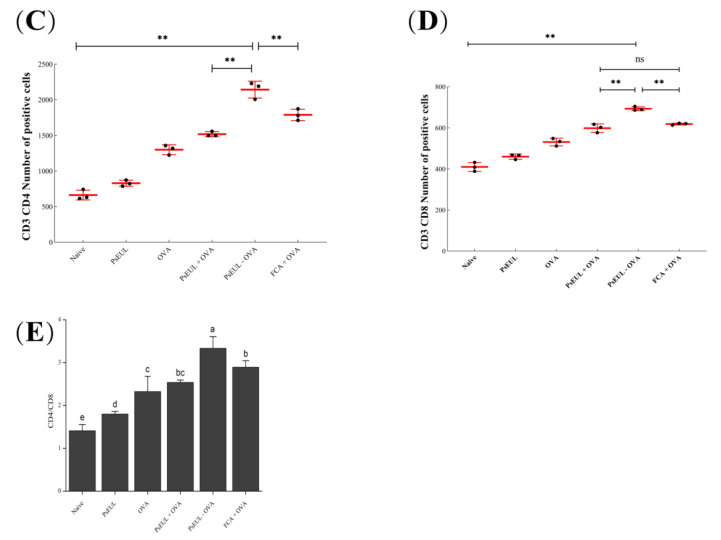
Effects of PsEUL-OVA on the phenotype of spleen lymphocytes in immunized mice. Samples were collected 28 days after the first immunization. (**A**,**B**) shows a point diagram of the T lymphocyte subpopulation double-stained with CD3/CD4 and CD3/CD8. (**C**,**D**) shows CD4^+^ and CD8^+^ T cell number distribution maps, and significant differences are designated as ** *p* < 0.01. “ns” represents no significant difference. (**E**) shows the ratio of CD4^+^ T cells to CD8^+^ T cells; results are presented as mean ± SD (*n* = 3). Different letters (a–e) above each group of bars indicate statistically significant differences (*p* < 0.05).

CD3^+^ is the surface marker of mature T lymphocytes in the body. CD4^+^ T lymphocytes are also called Th cells. CD8^+^ T lymphocytes are also called suppressor T(TS) cells. CD4^+^ and CD8^+^ T cells, using labeled CD3^+^ cells, can filter out mature CD4^+^ T and CD8^+^ T lymphocytes, respectively. The ratio of CD4/CD8 reflects the body’s immune function [54,55,56]. It can be seen in Figure 9A,C that the expression of CD4^+^ T lymphocytes in the PsEUL-OVA group is significantly higher than that in the FCA+OVA, PsEUL+OVA, and naive groups (*p* < 0.01). The levels of CD8^+^ T lymphocytes of mice in the PsEUL-OVA group were significantly higher than those of mice in the other groups (Figure 9B,D, *p* < 0.01). The CD4/CD8 ratio in the PsEUL-OVA group was significantly higher than that in the other groups (Figure 9E, *p* < 0.01). Data from the present study suggested that PsEUL-OVA could induce a stronger cellular immune response in immunized mice and also demonstrated that PsEUL could function as an adjuvant conjugated with OVA and induce a Th1-biased immune response in mice.

### 3.9. Effects of PsEUL-OVA on the Maturation of DCs in ICR Mice

**Figure 10:**
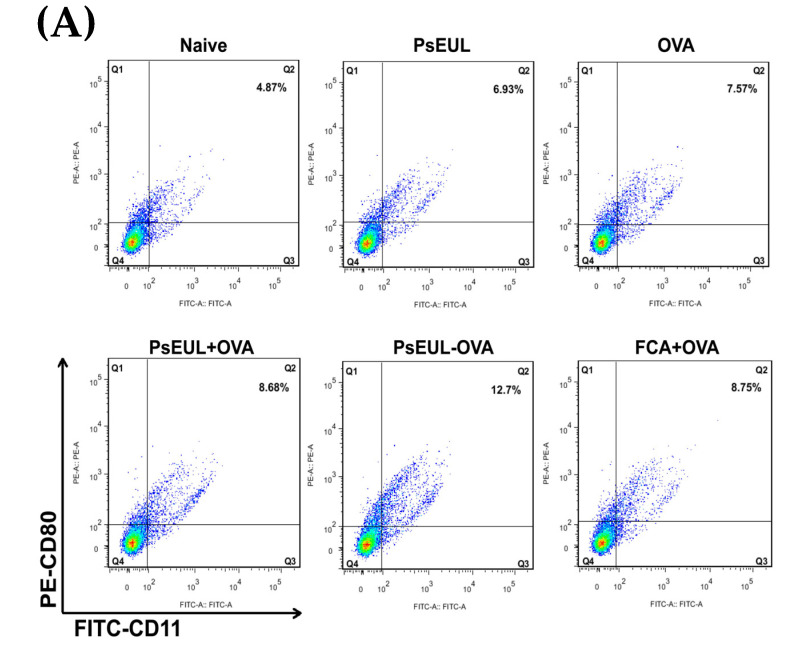

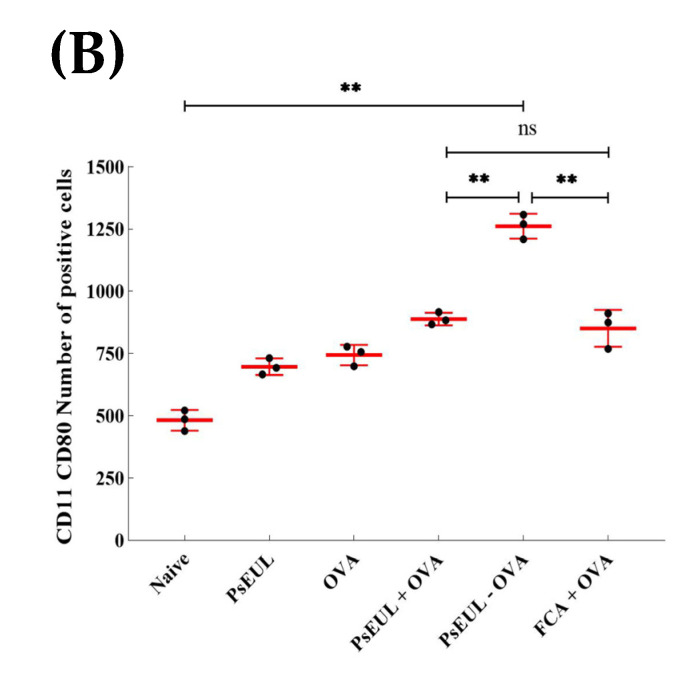

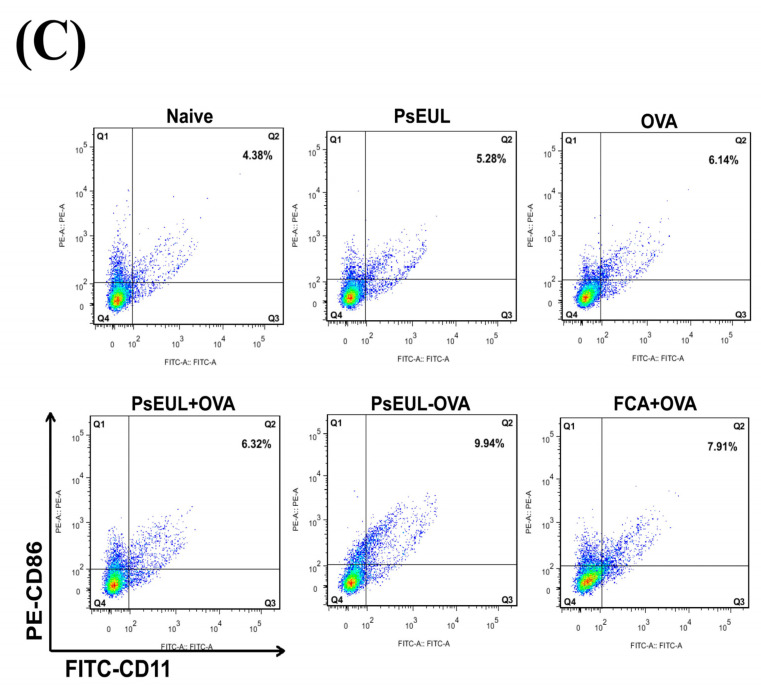

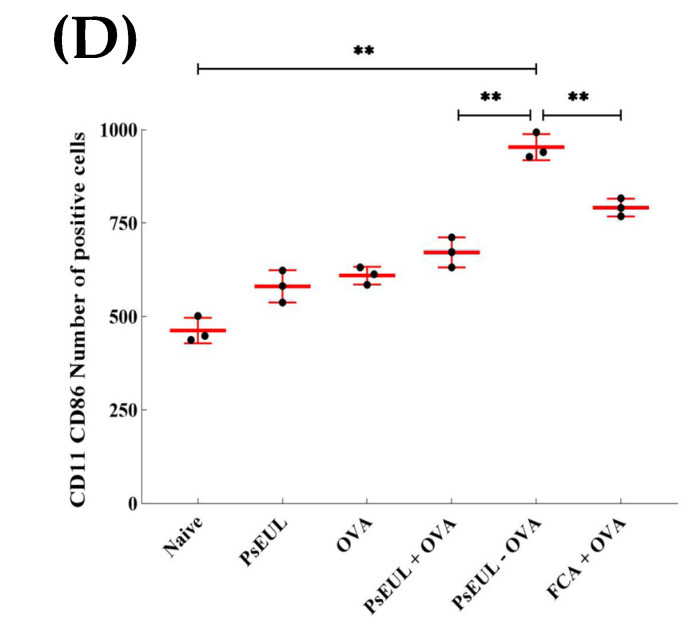

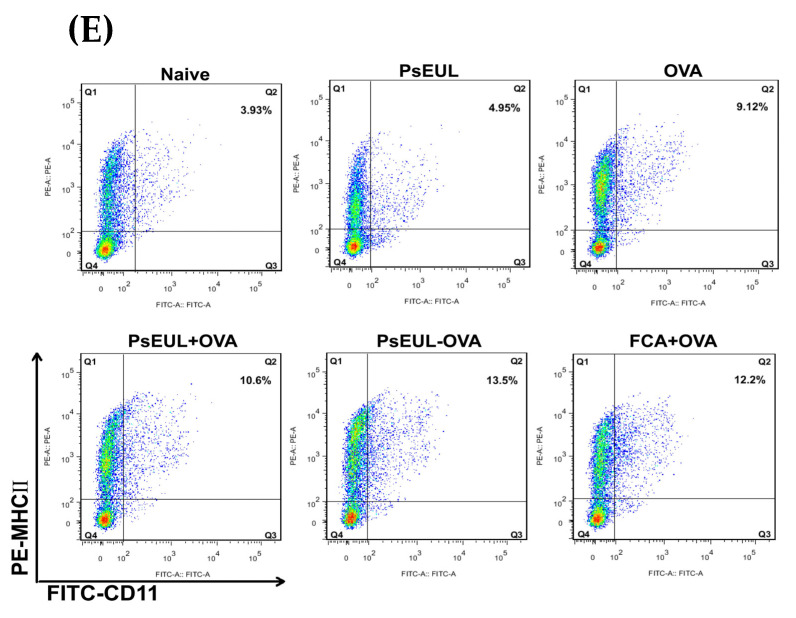

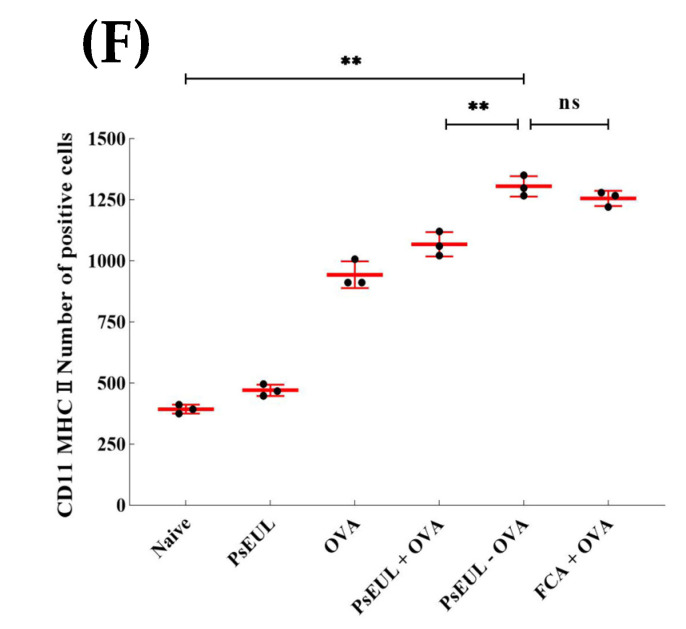
Effects of PsEUL-OVA on the maturation of DCs in ICR mice. Total splenocytes were isolated from the spleens of ICR mice 3 days after the first immunization. Point diagram analysis of dendritic cells double-stained with CD11c-CD80, CD11c-CD86, and CD11c-MHCⅡ (**A**,**C**,**E**). Distribution map of CD11c-CD80-, CD11c-CD86-, and CD11c-MHCⅡ-co-expressing cells (**B**,**D**,**F**). Significant differences between the PsEUL-OVA groups were designated as ** *p* < 0.01, and “ns” represents no significant difference.

The maturation of dendritic cells can reflect the level of cellular immune response and can be evaluated by determining the expression levels of CD80^+^, CD86^+^, and MHCⅡ in dendritic cells [57]. As illustrated in Figure 10A,B, the expression level of CD80^+^ in the PsEUL-OVA group was significantly higher than that in the FCA+OVA, PsEUL+OVA, and naive groups (*p* < 0.01). The expression level of CD86^+^ in the PsEUL-OVA group was significantly higher than that in all other groups (*p* < 0.01, Figure 10C,D). As seen in Figure 10E,F, the expression level of MHCⅡ in the PsEUL-OVA group was significantly higher than that in the PsEUL+OVA and naive groups (*p* < 0.01). Results from the present study indicated that PsEUL-OVA could increase the maturation of DCs.

## 4. Discussion

In recent years, polysaccharide-conjugated delivery systems have gained much attention in the field of applied materials science, and several studies have shown that polysaccharide modifications can greatly increase antigens’ immune responses [30,31]. In the present study, our findings demonstrate that PsEUL-OVA efficiently improved antigen internalization and upregulated the proliferation response in vitro. In vivo, PsEUL-OVA induced the maturation of dendritic cells and the expression of CD4^+^ and CD8^+^ on spleen lymphocytes, and maintained the CD4^+^/CD8^+^ balance. Moreover, PsEUL-OVA stimulated Th1/Th2 cytokine and antigen-specific IgG and IgG subclass antibody production. Our results reveal that PsEUL-OVA efficiently delivered the OVA to DCs and significantly promoted antigen-presenting efficiency.

The EDC condensation reaction is a widely used method that can activate proteins. When chemically bonded to polysaccharides, synthetic products can retain the activity of proteins and polysaccharides to the greatest extent when absorbed by the body [25,26]. In the current study, the EDC method was used to couple PsEUL to OVA. Data from FT-IR (Figure 1) and SDS-PAGE (Figure 2) showed that the EDC method was successfully used to couple PsEUL and OVA. PsEUL in the third lane of SDS-PAGE showed no protein bands during electrophoresis, indicating that PsEUL was extracted using the Sevag method. This treatment completely removed the proteins in PsEUL and high-purity polysaccharides were obtained. By measuring the particle size and zeta potential of the PsEUL-OVA, we confirmed that PsEUL-OVA has high stability. This finding lays the foundation for subsequent drug trials.

Macrophages are monocytes with strong phagocytic ability, especially against pathogens [58]. Macrophages take up antigens and play an important role in the systemic immune response. Using the CCK-8 method to determine the effect of PsEUL-OVA on macrophage activity, we found that PsEUL-OVA, as a model antigen, was non-toxic to cells and could also enhance cell proliferation. This occurrence may have been due to PsEUL activating specific receptors on the macrophages, thereby activating the macrophages and leading to the acceleration of the cell cycle, which in turn led to macrophage proliferation. At a concentration of 200 μg mL^−1^, the activity of macrophages was found to be highest; therefore, this concentration was used for experiments related to cell phagocytosis. Results concerning the phagocytic efficiency of macrophages against antigens, observed using laser confocal scanning microscopy, revealed the improved ability of macrophages to phagocytose antigens in the presence of PsEUL. After coupling the antigen with PsEUL, macrophages could take up more antigens, likely due to specific polysaccharide receptors on the macrophage surface. A large amount of PsEUL-OVA was adsorbed on the cell surface of macrophages, which greatly increased the ability of macrophages to take up the antigen.

Immunoglobulin (Ig) is a protein secreted by effector B cells that can specifically bind to the corresponding antigen when the antigen stimulates the body’s immune system. Among these, IgG is the most common, and most antibody reactions involve IgG-mediated effector functions. The presence of specific IgG and its subclasses in serum reflects the body’s immune level and the expression level of different subclasses of IgG determine the type of immune response [51]. The production of OVA-specific IgG and specific IgG subclass in mouse serum was determined using ELISA. Our findings demonstrated that PsEUL-OVA could increase the level of OVA-specific antibodies and antibody subclasses in mouse serum. In general, Th1 and Th2 produce IgG2a and IgG1 antibodies, respectively. Therefore, the IgG2a/IgG1 ratio is commonly used to evaluate the immune response induced by vaccines [52]. Qiao et al. conjugated tetanus toxoid (TT) with meningococcal group Y CPS to produce conjugate antigen (CPS-TT), and CPS-TT was further conjugated with β-glucan to obtain a vaccine CPS-TT-G. Their findings have demonstrated that using β-glucan as an adjuvant conjugated with antigen (CPS-TT) can significantly promote CPS-specific antibody IgG titers [59]. In this study, it can be seen that this ratio in the OVA, PsEUL+OVA, PsEUL-OVA, and FCA groups was significantly higher than that in the naive and polysaccharide groups. Compared to the OVA group, the ratios in the PsEUL+OVA, PsEUL-OVA, and FCA groups were significantly increased. Our results suggested that PsEUL-OVA could induce a stronger humoral immune response and exhibited a Th1-type bias.

Cytokines are mainly small molecule polypeptides or glycoproteins synthesized and secreted by immune cells. Cytokines can mediate the interaction between cells and regulate the immune response, including the production of interleukins (IL), interferons (IFN), and tumor necrosis factor (TNF) [60,61,62]. Th0 cells can form Th1 cells after encountering IL-12. Th1 cells mainly secrete IL-2 and INF-γ, whereas Th2 cells mainly secrete IL-4 and IL-6. IL-4 can induce Th0 cells to differentiate into Th2 cells. IL-4 can inhibit the expression of IL-12, thereby preventing Th0 cells from differentiating into Th1 cells. Th1 and Th2 cells are important immune cells in the body. Th1 cells are biased toward cellular immunity and Th2 are biased toward humoral immunity. Therefore, Th1 and Th2 cells are always maintained in a relatively balanced state in the body. An interruption in this balance indicates that the body’s immune system is affected. The Th1/Th2 ratio is commonly used in medicine to assess the state of the body’s immune system [63]. During immunization, the body’s immune system responds accordingly because antigens are foreign substances. Studies dealing with vaccine research show that most of the immune reactions that occur during vaccination are mainly based on cellular immunity [64], that is, the Th1-type response increases. Therefore, the level of serum cytokines is often used to reflect the body’s immune activity and immune response bias.

Huang et al. confirmed that the Arabinogalactan-poly (I:C) (AG-P) was conjugated with Ag85B-HspX fusion protein (AH) to generate a vaccine (AH-AG-P) that can induce cytokine (IFN-γ, TNF-α, IL-2, and IL-4) production in C57BL/6 mice [65]. In the present study, PsEUL-OVA could increase the level of cytokines in mouse serum. Compared to the FCA+OVA group, the concentrations of IFN-γ and IL-2 in the PsEUL-OVA group were higher, whereas those of IL-4 and IL-6 were significantly lower. These findings indicated that PsEUL-OVA could increase the immune level of mice and bias the Th1 type response; our results were consistent with those obtained using serum-specific antibody isotypes.

T lymphocytes can be divided into CD4^+^ T cells and CD8^+^ T cell subsets. CD4^+^ T cells are important immune cells in the body’s immune system. CD4 is a T cell antigen receptor (TCR) that activates CD4^+^ T cells to secrete many different cytokines to regulate the function of cytotoxic T lymphocytes (CTL) and B cells. CD8 is also a Th cell TCR; CD8^+^ T cells differentiate into CTL after activation and can specifically kill target cells. Therefore, the CD4^+^ T/CD8^+^ T ratio is used clinically to reflect the activity of T lymphocytes [66]. Huang et al. demonstrated that the Ag85B-HspX fusion protein (AH) conjugated with Arabinogalactan-poly(I:C) (AG-P) to generate a vaccine (AH-AG-P) can significantly increase the proliferation and differentiation of CD8^+^ T cells in C57BL/6 mice [67]. In the present study, we measured the ratio of CD3^+^CD4^+^ and CD3^+^CD8^+^ double-positive cells and found that the number of CD4^+^/CD8^+^ T cells in the PsEUL-OVA group mice increased significantly, indicating that CD4^+^ T cells could proliferate after immunization with the PsEUL-OVA and regulate CTL activity.

DCs are currently the most powerful immune cells for antigen presentation. Immature DCs are induced into mature DCs under the action of co-stimulatory signaling molecules. CD80 is an important substance for T lymphocyte activation. Although CD86 can induce T lymphocytes to produce IL-2, MHCⅡ is a sign of the maturation of DCs [68]. In this study, the expression of co-stimulatory signaling molecules was evaluated using double-positive cells labeled with CD11c. Results showed that mice immunized with PsEUL-OVA could increase the expression of CD80, CD86, and MHCⅡ, revealing that PsEUL-OVA could significantly facilitate the maturation of DCs.

To date, vaccination is the most effective way to control infectious diseases. The antigen delivery systems or adjuvants are required to promote immune response production or increase the effectiveness of vaccines [69]. Some natural polymers with antiviral activity have good potential as antiviral agents, adjuvants or delivery systems for antiviral drugs and vaccine formulation development [70]. In the current study, PsEUL conjugated OVA, used to generate a delivery system, could be an efficient approach for antigen presentation that induces stronger antigen-specific immune responses than free antigens. Therefore, PsEUL may have potential as SARS-CoV-2 virus antigen delivery systems for optimal antigen-specific immune responses of vaccines.

In summary, we have demonstrated the feasibility of an antigen delivery system, PsEUL-OVA, in delivering OVA to macrophages and DCs. PsEUL-OVA significantly induced cellular and humoral immune responses by facilitating DC maturation. This PsEUL-based delivery system showed strong immunomodulation efficiency in promoting antigen presentation and activating T cells, and it could be applied to improve the effectiveness of new vaccines. However, the mechanism of this modulation effect of the PsEUL-based delivery system needs to be further investigated. This study has created a platform for future research studies on adjuvant and antigen delivery and the prolongation of their efficacy.

## 5. Conclusions

Herein, for the first time in the field, we explored the chemical coupling of PsEUL and OVA to yield PsEUL-OVA. Our results indicated that PsEUL-OVA has good immune-adjuvant activity and could enhance immunogenicity. In vitro, PsEUL-OVA not only promoted the proliferation of macrophages but also enhanced the phagocytosis of macrophages. PsEUL-OVA could not only activate a stronger antigen-specific immune response and enhance cytokine levels in vivo, but also exhibited a Th1-type biased immune response that prompted the maturation of T lymphocytes and DCs. Taken together, the results of our study help us to understand the positive regulation of PsEUL-OVA in the immune system. These findings pave the way towards using PsEUL-based drug delivery systems for future vaccine formulations and delivering nanotherapeutics to diseased cells, improving their potency and efficacy. Future studies will further investigate the underlying mechanisms of the PsEUL-based drug delivery system in relatin to the immune response, and its potential application in other animal models. Our study shows the prospect of developing natural polysaccharide-conjugated antigen nanodelivery systems to improve the body’s immune response.

## Data Availability

Not applicable.
